# Structured Semantic Knowledge Can Emerge Automatically from Predicting Word Sequences in Child-Directed Speech

**DOI:** 10.3389/fpsyg.2018.00133

**Published:** 2018-02-22

**Authors:** Philip A. Huebner, Jon A. Willits

**Affiliations:** ^1^Interdepartmental Neuroscience Graduate Program, University of California, Riverside, Riverside, CA, United States; ^2^Department of Psychology, University of California, Riverside, Riverside, CA, United States

**Keywords:** semantic development, language learning, neural networks, statistical learning

## Abstract

Previous research has suggested that distributional learning mechanisms may contribute to the acquisition of semantic knowledge. However, distributional learning mechanisms, statistical learning, and contemporary “deep learning” approaches have been criticized for being incapable of learning the kind of abstract and structured knowledge that many think is required for acquisition of semantic knowledge. In this paper, we show that recurrent neural networks, trained on noisy naturalistic speech to children, do in fact learn what appears to be abstract and structured knowledge. We trained two types of recurrent neural networks (Simple Recurrent Network, and Long Short-Term Memory) to predict word sequences in a 5-million-word corpus of speech directed to children ages 0–3 years old, and assessed what semantic knowledge they acquired. We found that learned internal representations are encoding various abstract grammatical and semantic features that are useful for predicting word sequences. Assessing the organization of semantic knowledge in terms of the similarity structure, we found evidence of emergent categorical and hierarchical structure in both models. We found that the Long Short-term Memory (LSTM) and SRN are both learning very similar kinds of representations, but the LSTM achieved higher levels of performance on a quantitative evaluation. We also trained a non-recurrent neural network, Skip-gram, on the same input to compare our results to the state-of-the-art in machine learning. We found that Skip-gram achieves relatively similar performance to the LSTM, but is representing words more in terms of thematic compared to taxonomic relations, and we provide reasons why this might be the case. Our findings show that a learning system that derives abstract, distributed representations for the purpose of predicting sequential dependencies in naturalistic language may provide insight into emergence of many properties of the developing semantic system.

## Introduction

The development of semantic memory is an extremely complex phenomenon, requiring input from all perceptual modalities and making use of many psychological processes. Recent research efforts into this topic have focused on the *distributional hypothesis* (Harris, 1954; Firth, 1957), the claim that the similarity, class membership, or relations between linguistic units or concepts can be inferred from the statistical or structural contexts in which those units occur.

In the computational realm, this idea was formalized in a range of different models of adult semantics, such as Latent Semantic Analysis (LSA, Landauer and Dumais, 1997), the Hyperspace Analogue to Language (HAL, Lund and Burgess, 1996), Bound Encoding of the Aggregate Language Environment (BEAGLE, Jones and Mewhort, 2007), and Probabilistic Topic Models (Topics, Steyvers and Tenenbaum, 2005). These models use distributional information to construct semantic feature vectors for words. Feature vectors can be composed of concrete associations between words/concepts, as in the HAL model, or they can consist of abstract or latent features that are formed over the course of learning (as in the other three models). The semantic similarity of two words can then be calculated by measuring the similarity of the two words’ feature vectors (Kahneman and Tversky, 1972; Smith et al., 1974). Considerable research has since shown that these and related procedures for representing semantic similarity predict a wide range of adult psycholinguistic variables, such as semantic priming and explicit similarity judgments (Burgess and Lund, 1998; Jones et al., 2006; Bullinaria and Levy, 2007; Olney et al., 2012; Pereira et al., 2016).

Concurrent to work in computational modeling of semantic memory, researchers in child language acquisition were studying whether children are sensitive to distributional information, and whether they can use it to infer word meanings. Gleitman (1990) suggested that syntactic bootstrapping (i.e., inferring aspects of a word’s meaning from its syntactic structure) may be an important mechanism by which children begin to learn the meanings of words. Using syntactic bootstrapping, children may, for example, infer whether a verb is transitive or intransitive by tracking whether the verb occurs with one or two nouns or noun phrases. Recent studies have shown that infants and children are sensitive to the distributional structure of words, and do seem to infer aspects of word meaning from lexical and syntactic distributional structure (Fisher et al., 2010; Lany and Saffran, 2010; Syrett and Lidz, 2010; Wojcik and Saffran, 2013).

Thus, both computational and experimental work has shown that substantial semantic information exists in words’ distributions, *and* that human learners are sensitive to this information. However, the distributional hypothesis leaves many questions unanswered, and each can be traced back to three theoretical debates in language and concept acquisition.

The first debate concerns the *abstractness* of knowledge: Does knowledge consists primarily (or exclusively) of a rich sets of associations between sensory-motor features, or instead also consists of abstract, amodal concepts that bind those features together? This is a very old question, made very salient by the debate between Chomsky (1957, 1959) and Skinner (2014) about the nature of language, and between Osgood (1952, 1966) and Fodor (1965) in the study of semantics. Recent research demonstrating children’s powerful statistical learning abilities (Saffran et al., 1996; Sloutsky, 2003; Smith and Yu, 2008) and the power of statistical machine learning approaches (Hinton et al., 2012; Mikolov et al., 2013a; Chen and Manning, 2014) have revived interest in statistical learning-based approaches, but persuasive theoretical arguments for abstract concepts still exist. Waxman and Gelman (2009) succinctly describe this as a debate between two metaphors. The first is “*child as data analyst*,” whereby language acquisition occurs because of children’s amazing statistical learning skills and their ability to build webs of associations of a wide variety of perceptual inputs and motor actions. This is contrasted with the “*child as theorist*” metaphor, whereby children begin with and/or build up theories about the world involving rich conceptual knowledge structures, and these knowledge structures play a critical role in structuring language acquisition. Waxman and Gelman accept a role for statistical learning, but reject an exclusively “*child as data analyst*” perspective, arguing that abstract concepts play a critical role in language acquisition and knowledge representation.

The second debate concerns the *organization* of knowledge. Is knowledge an unstructured set of associations, or is knowledge instead stored in structured and hierarchically-organized representations? In language, this debate is often characterized as whether an utterance or sentence is best understood as a set of associations between the constituent elements, or instead as some kind of structured, tree-like representation (Chomsky, 1957; Miller, 1962). In semantics, this debate is often characterized as whether knowledge is organized in a hierarchically embedded graph structure (Katz and Fodor, 1963; Collins and Quillian, 1969; Anderson and Stageberg, 1975), or is instead stored in a more disordered set of associations (Collins and Loftus, 1975; Rosch, 1975). For many researchers, a host of behavioral phenomena in linguistic and conceptual behavior (such as long distance dependencies in language, and patterns of induction and generalization in semantics) suggest some form of hierarchical representations are necessary.

The distinction between the first and second debates is often confused but is very important, creating (in principle) four distinct theoretical positions as a function of one’s view of the form of knowledge (knowledge of associations of concrete sensory-motor features vs. abstract concepts) and the organization of that knowledge (structured and hierarchical vs. unstructured). These independent theoretical positions are summarized in **Table 1**.

**Table 1:** Theoretical debates regarding the nature of knowledge.

	Form of knowledge	
Structure of knowledge	Only representations of sensory-motor information	Sensory-motor and abstract concepts
Unstructured	Knowledge consists of nothing but a very rich and complex set of unstructured sensory-motor associations. Example: Sloutsky and Fisher, 2004.	In addition to sensory-motor information, knowledge consists of a set of abstract concepts. Example: Collins and Loftus, 1975.
Structured	Knowledge consists of a very rich and complex set of sensory/motor associations that are organized into hierarchically-structured representations. Example: Most neural theories of sensory-motor behavior	Knowledge consists of sensory/motor information and abstract concepts, organized into hierarchically-structured representations. Example: Tenenbaum et al., 2011

It is difficult, if not impossible, to clearly associate the distributional hypothesis with any single theoretical position, as distributional models are often criticized by proponents of all four. In this paper, we emphasize that, while specific models instantiating the distributional hypothesis typically take a position on these theoretical debates (and thus open themselves up to criticism from those who disagree), the distributional hypothesis, *per se*, is orthogonal to these debates. For example, neural networks, as statistical learning algorithms, are often lumped into what Waxman and Gelman call “child-as-data-scientist” explanations. But most neural network models that include “hidden layers” (e.g., layers of units that involve compression, re-representation), are indeed representing abstract concepts, even if they are not the same ones Waxman and Gelman would suggest.

Likewise, neural network models are often criticized for not representing language or concepts in a hierarchical way that is necessary for language (Fodor and Pylyshyn, 1988; Pinker and Prince, 1988; Marcus, 1998; Gershman and Tenenbaum, 2015). But it is useful to distinguish here between what a neural network can represent, and what a neural network can *learn* to represent. Any structured, hierarchical representation can be encoded in a vector representation, and can be represented in a network’s weights. Neural networks with hidden layers are, after all, universal function approximators. Thus, there is nothing about neural networks that is incompatible with a theory that says that language must be represented as a system of discrete, hierarchically-organized symbols. The question is whether any particular neural network model can learn the correct structured representation of the language from the input.

State-of-the-art neural network models excel at mapping a sequence of words to its corresponding syntactic structure (Chen and Manning, 2014), but these models need to be supplied with the set of possible syntactic structures in order to do so, and have trouble learning those structures from the ground up. Some success has been achieved by Rogers et al. (2004) and Rogers and McClelland (2008), who showed that a feedforward neural network, learning about concepts in terms of the correlational structure of their shared features or propositional content (such as *canaries* “*are yellow*” and “*have wings*”) can be used to explain the apparent hierarchical nature of concepts, and argued that hierarchical-like behavior is an emergent property of distributed representations representing the relative similarity of concepts. However, critics of Rogers and McClelland have argued that their model used a simplistic, idealized view of what children’s input is actually like. They claim that the “poverty of the stimulus” would prevent such a model from explaining real-world semantic development (Marcus and Keil, 2008; Robbins, 2008; Snedeker, 2008).

Our paper is designed to specifically address whether three specific neural network architectures that instantiate the distributional hypothesis are capable of acquiring useful organization of semantic knowledge from complex, noisy, naturalistic input and to assess the extent to which the knowledge these networks have acquired is abstract and hierarchically-organized. We employed two recurrent artificial neural network models – the Simple Recurrent Network (SRN) (Elman, 1990) and the Long Short-term Memory (LSTM) (Hochreiter and Schmidhuber, 1997) – and a third, Skip-gram, which is one variant of the Word2Vec family of models (Mikolov et al., 2013a). These models learn representations of words by predicting a word given a context and updating model parameters to minimize the prediction error. A feed-forward neural network can be used to predict a word given its co-occurrence context, and the resulting representations the network learns in order to do this contain surprisingly rich semantic information (Bengio et al., 2003; Mikolov et al., 2013a; Pennington et al., 2014). The most popular of prediction-based models, a family of models often referred to as Word2Vec (Mikolov et al., 2013a), has become a popular off-the-shelf tool for learning word representations from text in machine learning applications. The representations learned by models in the Word2Vec family (such as Skip-gram, the algorithm we will focus on in this paper) outperform a number of publicly available word representations in a benchmark test that includes 8869 semantic and 10675 syntactic questions (Mikolov et al., 2013a).

The Skip-gram model raises some concerns with regards to being taken seriously as cognitively plausible models of semantic development. For example, Word2Vec implementations contain a number of optimizations to speed training on large corpora, but some of these optimizations seem unlikely to be the way the human brain performs prediction-based learning. One requirement for training Word2Vec’s Skip-gram model is knowing beforehand the frequency of words in the corpus (such that relatively frequent words can be downsampled), knowledge that is inaccessible in online learning circumstances. Another concern is Skip-gram’s negative sampling procedure (Mikolov et al., 2013b), where for each prediction, only a subset of possible words are sampled from the vocabulary, including the correct next word, and others drawn from a distribution that does not include the correct word. This procedure requires knowing the correct prediction before the outcome of the prediction is computed. While this speeds training and increases performance in a machine learning context, there is no evidence for such a complex memory-based process in online human learning. A number of other optimizations (such as using the current word to “postdict” previous words in the stream) have no current basis in theories of human language processing, though this of course does not mean that such processes are impossible.

There are other prediction-based neural networks that might serve as more plausible candidates for theories of semantic knowledge acquisition than Word2Vec. For example, the first studies using Simple Recurrent Networks (SRNs) showed they could learn to predict sequences, and that doing so enables learning about the structure of the items in those sequences (Elman, 1990, 1991; Cleeremans and McClelland, 1991). For example, Elman (1991) showed that the SRN could learn the regularities of an artificial linguistic corpus composed of thousands of sentences constructed following an extremely simplified English grammar composed of nouns, verbs, articles, and prepositions. Elman showed that the SRN could learn to predict the “correct” words in terms of following the grammatical rules and semantic constraints that were used to generate the corpus, such as noun-verb number agreement, even in cases where the verb was separated from the noun by multiple embedded clauses. Furthermore, its ability to track number agreement diminished as the length of intervening words grew larger, and this reflects experimental observations in humans.

The SRN’s success at this task was due to its ability to compress sequential information into a compact distributed representation in the hidden layer. In a distributed representation, a concept is represented by a pattern of activations across an ensemble of units; by design, no single unit can convey that concept on its own. Elman showed that the similarity structure between the learned distributed representations can be interpreted as a measure of grammatical and semantic similarity between the words they represent. However, like previous researchers investigating feedforward models, Elman used an artificial and simplified corpus, and therefore left open the question of whether the SRN can scale up to noisy naturalistic language input. Recent large-scale language modeling efforts using written language corpora suggest that SRNs can reach prediction performance equal to, and in some cases surpassing, n-gram models (until recently the most widely used language modeling tool, and now largely replaced by neural networks, Mikolov et al., 2014), but its success on noisy naturalistic language input, such as speech to children has not been investigated.

A third model which we consider in this paper is the Long Short-term Memory (LSTM) model (Hochreiter and Schmidhuber, 1997). In this paper, we will use LSTM to refer to a network with a recurrent hidden and output layer, where conventional hidden layer units are replaced by LSTM units. As we shall describe below, the LSTM units differ from the traditional hidden units found in the SRN by their ability to regulate the flow of information to and from themselves. This added machinery greatly increases learning of long sequences. The LSTM is of interest because it has proved successful on a variety of sequence modeling tasks such as learning context free languages (Gers and Schmidhuber, 2001), and recalling high precision real numbers over long and noisy sequences (Hochreiter and Schmidhuber, 1997), tasks which are very difficult, if not impossible, for the SRN to learn. Furthermore, the LSTM reached substantially greater accuracy on a variety of number agreement tasks compared to the SRN (Linzen et al., 2016), and was used recently by Microsoft Research to reach human parity in conversational speech recognition (Xiong et al., 2016). By training both the SRN and LSTM on the same input, we can get an idea of how the ability to track sequential dependencies might influence the semantic structure that emerges. If differences are observed, they will be due to the specific architectural improvements of the LSTM relative to the SRN, and as such will provide insight into the design of a model of semantic development.

In this paper, we use three different neural networks to test the distributional hypothesis, and to ask three specific questions about the relationship between these models and the distributional hypothesis. First, can the neural networks learn semantic structure from predicting the word sequences of noisy, naturalistic speech to children? Second, if so, do the semantic structures that the models acquire reflect the semantic structures that children acquire? Third, do different neural network architectures (and the different theories of learning and memory that they represent) perform qualitatively or quantitatively differently?

## Materials and Methods

### Corpus

As noted above, one major criticism of previous work showing that neural networks learn abstract and highly structured knowledge is that these demonstrations have tended to use small, artificial datasets that do not capture the real noise and complexity of speech to children. To address this problem we trained our models on the CHILDES corpus, a collection of transcripts of interactions with children in various situations (MacWhinney, 2000). CHILDES contains a mixture of transcriptions of structured in-lab activities (such as book-reading, mealtime, and playing with toys), free play in the lab, and in-home recordings.

We used all transcripts involving typically-developing children 0–3 years of age from American–English-speaking households. This resulted in a corpus containing 2873 documents, 22,448 word types, and 5,308,679 word tokens, collected from 52 different studies of parent–child interactions. We randomly split the documents into separate training (5,244,672 word tokens) and testing (64,007 word tokens) corpora, where the former will be used for training, and the latter will be used to assess generalization to input not encountered during training. Considering that a typical working-class American child receives approximately 6.5 million words per year (Hart and Risley, 2003), the training corpus represents approximately 4–10% of the amount of lexical input of a 3-year-old child (there are large individual differences largely predictable by socio-economic status). The documents of the corpus were organized by the age of the child spoken to, such that each model experienced the input in an age-appropriate way, receiving the input a 6-month-old hears, then a 7-month-old, then an 8-month-old, etc.

The transcribed corpus was tokenized (split on spaces) with sentence-boundary punctuation (periods, exclamation marks, commas, and question marks) left in the corpus (intended to serve as a very crude way for representing the pauses and prosody that tend to accompany utterance boundaries). Spelling was regularized (e.g., differently spelled forms of the same word like “*play-doh*” and “*playdoh*” were converted to the same form). Next, all nouns and verbs in the corpus were morphologically parsed, splitting off plural (-*s*, -*es*), possessive (-*’s*, -*s’*), and diminutive forms (-*ie* and -*y*) from nouns, and splitting off plural (-*s*,–*es*), past-tense (-*ed*) and ongoing (-*ing*) forms from verbs. These morphological endings were left in the corpus (effectively treating them as words of their own), due to previous research showing a beneficial effect on statistical learning models of natural language (Willits et al., 2009). Proper names were replaced with tokens signifying the gender of the person in question (FNAME and MNAME).

CHILDES is not perfect as a representative sample of the full range of activities that parents participate in with their children or the variety of language used during those activities, but is instead a useful approximation. Indeed, the relatively constrained set of activities that occur in CHILDES ought to hinder learning of useful semantic structure, and thus make positive results all the more impressive.

### Vocabulary, Probe-Words, and Categories

To reduce training time and simulate the fact that children are unlikely to know the lexical form of the lowest frequency items in the corpus, we limited the model’s vocabulary to the 4096 most frequent word types. The other 18,352 word types (81% of total word types in the corpus) were replaced with the symbol UNKNOWN. Given that word distribution obeys a power law, only 0.79% of all total word tokens (41,532 out of 5,308,679) in the corpus were affected. In an offline analysis, we included all words that occur at least twice (12,511 word types in the vocabulary) and found no improvement in learning outcomes.

In order to address the question of whether the model was learning abstract and structured knowledge, we chose to investigate the model’s knowledge of a set of probe words belonging to a set of pre-identified categories. We selected a subset of the vocabulary words to serve as probe words for all subsequent analyses by, (1) choosing the subset of word forms which could be nouns (even if, in practice they appear more often in verb form, such as *jump*), (2) choosing the subset of those that refer to a concrete object, and (3) choosing the subset of those that unambiguously belong to a semantic category from which at least six other words belong, according to a set of human raters. For example, *apple*, *orange*, and *banana* (along with many other fruit words) were included because they belonged to a large category of items that contained at least six items. The result was a set of 720 words belonging to 29 categories. This set of 29 categories used for and analyzing the models is shown in **Table 2**.

**Table 2:** The set of categories, the number of word types in each category, and the number of occurrences of word types in each category in the training corpus.

Category	Word types	Word tokens	Category	Word types	Word tokens
Bathroom	22	5533	Mammal	72	35781
Bird	27	8384	Meat	18	2914
Body	62	42601	Months	13	1897
Clothing	48	16022	Music	14	1845
Days	14	8163	Numbers	27	41048
Dessert	20	9048	Plants	15	6006
Drink	14	9880	Shape	13	3355
Electronics	18	5347	Space	14	3042
Family	32	52539	Times	11	7731
Fruit	28	7719	Tools	28	7665
Furniture	28	11131	Toys	30	25339
Games	6	1222	Vegetable	21	3271
Household	32	10930	Vehicles	34	15559
Insect	18	4755	Weather	11	4082
Kitchen	29	7767			

### Model Implementation

All three models were trained on a machine with 32 GB of RAM, an 8-core 3.0GHz Intel Xeon processor, and a NVIDIA GTX 1080 GPU. To train the recurrent neural networks, we used the open-source machine-learning framework TensorFlow (Abadi et al., 2016), and to train Skip-gram, we used Gensim (Rehurek and Sojka, 2010), a free Python library which provides APIs for a wide variety of semantic models. The code, including the training corpus, and test materials are available at https://github.com/phueb/rnnlab. Using a mini-batch size of 64, training one LSTM and one SRN on the GPU takes approximately 3 and 2.5 h, respectively. Using 4 CPU cores in parallel, Skip-gram completed training in less than 5 min.

### Model Architecture and Training

#### Simple Recurrent Network Architecture

The Simple Recurrent Network (SRN) is an artificial neural network that contains an input, a hidden, and an output layer, in addition to copy connections linking the hidden layer to the input layer at the next time step (Elman, 1990). The hidden layer learns distributed internal representations of the input, and the recurrent connectivity allows these representations to encode information from previous time steps. This means that the hidden layer’s pattern of activations is not a simple representation of the input stimulus, but rather the input stimulus in the context in which it occurred.

**FIGURE 1:**
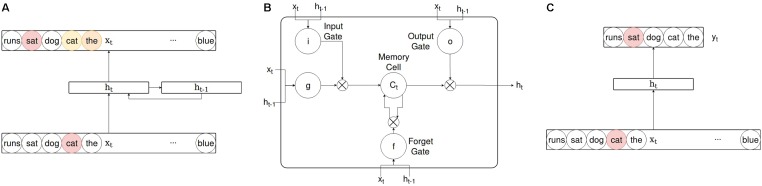
A simple comparison of model architectures showing the Simple Recurrent Network **(A)**, a single Long Short-term Memory (LSTM) unit **(B)**, and Word2Vec’s Skip-gram **(C)**. Note that 1B depicts a single LSTM unit, rather than the full LSTM model. The complete LSTM architecture consists of three layers as in **(A)** but with LSTM units instead of conventional sigmoidal or hyperbolic tangent units at the hidden layer. Colors depict the level of activation from 0 to 1 (yellow to red). Skip-gram does not have the full vocabulary represented at the output layer as in **(A)**, as Skip-gram is typically trained using negative sampling, where the objective is to predict the correct answer amongst a small selection of “negative” words.

A schematic of the SRN’s architecture is shown in **Figure 1A**. For each time step, the SRN received as input a localist representation of a single word drawn sequentially from the training corpus. The localist representational scheme ensures the model has no access to information about word similarity (phonological, semantic, etc.) at this stage. This is done by filling the input vector with zeros at every of 4096 positions (corresponding to the vocabulary size) except for the position uniquely assigned to the current input word. The goal of this scheme is not to claim that children do not utilize additional sources of information about input words, but to test just how rich a child’s semantic knowledge could become based on lexical distributional information alone.

The activations at the hidden layer (512 units) are the result of multiplying each input unit’s activation by the weighted connections from that input unit to each hidden unit. Critically, the pattern of activations from the hidden units at the previous time step is added, weighted by the recurrent connections. Lastly, each hidden unit activation is transformed by the hyperbolic tangent non-linearity to constrain its activation between -1 and +1.

The hidden layer activations are then sent via a third set of weighted connections to the output layer (again containing 4096 units). The net input into each output unit is first transformed by exponentiating *e* to the weighted input (effectively flattening the distribution. of activations across the output units). These output activations are then transformed into a posterior probability distribution of predictions of next most likely word in the corpus, given the input word. This is done using the softmax operation (dividing each output activation by the sum of all output activations).

#### Long Short-term Memory Architecture

Because the amount of information in the hidden layer representing distant past information decreases with each time step, learning dependencies across longer distances becomes increasingly difficult for the SRN. This problem has been referred to as “the vanishing gradient problem” (Hochreiter et al., 2001), referring to the vanishing across time steps of the signal carrying information that specifies how to update the weighted connections. Numerous workarounds have been suggested, and the most successful of them is the Long Short-term Memory (LSTM) unit introduced by Hochreiter and Schmidhuber (1997). The LSTM unit, rather than being a single unit, refers to three multiplicative gating units which control the flow of information to and from a central unit (termed the “memory cell”) whose activation does not undergo non-linear transformation. The architectural details of a single LSTM unit are shown in **Figure 1B**. Note that this is not the complete LSTM; instead, we will refer to the LSTM as a three-layer neural network similar to the SRN, with LSTM units replacing conventional hidden layer units.

To illustrate the advantage of the gating units, consider that in the SRN (which does not employ gating units), long-term memory is stored in the hidden layer where new information is integrated with little regard to whether it is relevant to the model’s objective or might instead mask already existing (and possibly useful) information. To prevent this, long-term information in the LSTM is stored in the memory cell where read and write access are a function of the input and forget gate unit, respectively. More precisely, incoming information can only be added to the memory cell if the input gate is not set to zero, and this can prevent integration of information that might not be useful. Similarly, if information already contained in the memory cell at the previous time step is not useful at the next time step, the forget unit can flush the contents of the memory cell by being set to zero. The output unit then gates the content of the memory cell after being transformed by a non-linear function (hyperbolic tangent), and it is the output of this gating operation which is typically fed into the output layer. Thus, the LSTM unit contains two distinct outputs which are reused across time steps: the content of the memory cell, and the result of the output gating operation. The former can preserve gradient information across more time steps relative to conventional units due to preserving only the information that is most useful and because it does not undergo any non-linear transformation, while the latter allows for learning of non-linear relationships. The gating units receive information in the input and from the previous output, which are multiplied by a unique set of weights, and are then squashed through the sigmoid function to result in an activation bounded between 0 and 1. Because the weighted connections to the gating units are trainable, the network can learn to modify the flow of information into and out of the memory cell, and it will do so independently at every time step.

#### Recurrent Neural Network Training Regime

Contrary to conventional training methodology in which input sequences are presented to the model from multiple iterations over the whole corpus (epoch training), our models iterated over small partitions of the age-ordered corpus. As a simplified example, consider a corpus consisting of three partitions, each representing a day’s worth of input: the first partition was input to a 1-year-old, the second to a 2-year-old, and the third to a 3-year old. A typical neural training procedure that iterated over the input five times would have the partitions presented to the model in the order: 1,2,3,1,2,3,1,2,3,1,2,3. Scaled to an entire dataset (with thousands of partitions/days of input), this is obviously a bit unrealistic, as it suggests a perfectly veridical memory of all items such that they can be successively iterated over. To address this, we instead presented the partitions in the order 1,1,1,1,2,2,2,2,3,3,3,3, simulating successive iterations of the same day’s input as model of memory consolidation of that day’s experiences. There are at least two potential advantages of this approach: first, given that our corpus contains transcribed speech ordered by the age of the children addressed, our models are sensitive to any change over time in the structure of child-directed speech, and we can investigate developmental trends and trajectories. Secondly, local iterations lend cognitive plausibility to our training regime because it is more likely that children consolidate linguistic experiences across time periods spanning hours or days rather than months or years. We split the training corpus into 256 partitions, so that each partition contains exactly 20,487 words, approximately the average number of words heard by children in 1 day (Hart and Risley, 2003). We trained 10 SRNs and 10 LSTMs using the same hyperparameters, but a different random seed during weight initialization. Weights were initialized with a truncated normal distribution with mean zero and standard deviation $1/\sqrt{m}$, where *m* is the number of units in the layer above. A bias unit was used at the output layer and its weights were initialized to zero. For every word in the training corpus, we feed into the model a sequence consisting of the word and the six words immediately left of it. The input was fed through the model (as described above) and resulted in a probability distribution of predictions for the next word in the sequence. We used the cross-entropy operation to compare a model’s predictions to the correct answer, which is equivalent to the negative log of the probability assigned by a model to the correct answer (i.e., the next word in the sequence). We used truncated backpropagation through time (Werbos, 1990; Williams and Peng, 1990) to compute the partial derivative of each layer’s activations with respect to the weights, and used these to update the weights in the direction that minimized prediction errors. This procedure was followed sequentially for each input sequence. We set the learning rate to 0.01 and used Adagrad optimization (Duchi et al., 2011) to adapt the learning rate so that infrequently changed weights received a greater update than those changed more frequently. Weights were adjusted using mini-batch training, in which weight updates only occurred after the accumulation of prediction errors from 64 words. In this way, the weight update reflects the average prediction error computed for all 64 sequences in the mini-batch. While the primary motivation for using mini-batching is to speed model training, the cognitive and neural plausibility of mini-batch learning is contestable. To address these concerns, we tested a range of different mini-batch sizes, finding that sizes greater than 64 led to slightly worse results, with no noticeable differences (other than in training time) for smaller mini-batch sizes, including a size of 1. Thus, we believe this detail in the model to be a benefit with regard to training time without a cost in terms of qualitatively or quantitatively changing the model’s behavior and thus calling into question its cognitive or neural plausibility.

#### Word2Vec’s Skip-Gram Architecture and Training Regime

Skip-gram is one of two members of the Word2Vec family of neural networks introduced by Mikolov et al. (2013a) as an efficient solution for obtaining word representations from very large corpora. Word2Vec models consist of an input, hidden, and output layer, and weighted connections to and from the hidden layer. While Word2Vec’s CBOW model generates word representations by predicting a word from its context, Skip-gram predicts the context from the word in which it occurred (shown in **Figure 1C**). We opted for Skip-gram because it produced superior results compared to CBOW on our corpus. In contrast to the LSTM and SRN, both Word2Vec models lack recurrent connections, and therefore do not learn contextual word representations (one for each occurrence of a word), or the precise order of words in the corpus.

One reason for including Skip-gram in a paper about child language learning is to compare the representations learned by the SRN and LSTM to those generated by the current state-of-the-art model in machine learning. Skip-gram’s performance will help to contextualize our results when making inferences about the SRN’s achievements. Secondly, and perhaps more importantly, because Skip-gram is not *explicitly* trained to learn word order (when Skip-gram predicts a word, it is not told what distance it was located away from the input word; however, it is trained more frequently on those that occur more closely), we can gain insight into how the degree to which information about word order during training influences learning of semantic structure. As described above, Skip-gram also includes a number of optimizations that increase its performance but vastly decrease its plausibility (or at least its parsimony) as a cognitive model. Despite these problems, we examined Skip-gram due to its popularity in the machine learning community as a semantic model, and to see if it varies in performance on our dataset or tasks.

We trained Skip-gram with a hidden layer size of 512, 20 epochs, and a bidirectional window size of 3. All other model properties were left as the default values specified by Gensim. Our choice of hyperparameters was driven by our goal of matching as closely as possible the hyperparameters used by the recurrent neural networks. Gensim did not provide the option of using the traditional softmax function at the output layer; so we were limited to using either hierarchical softmax or negative sampling. We opted for the hierarchical softmax as it is a more reliable estimate of the true softmax (Goldberg and Levy, 2014) which we employed in the LSTM and SRN. We found no difference in performance, however, when we used negative sampling.

## Results

Due to the simplicity and parsimony of the SRN compared to the other two models, we focus our analysis on its performance, and include results from the other two models only in cases where they differ substantially. We begin by confirming that the recurrent neural networks have learned the sequential structure of the language input. Next, we present analyses designed to assess the semantic knowledge that the models acquired through training.

### Sequential Structure Prediction

Before and after training, we calculated the average per-word perplexity, a measure of the model’s ability to correctly predict the next word in the sequence, on a subset of the CHILDES corpus not used during training (64,000 words). On a single prediction trial, the mean per-word perplexity score is equal to the number of times the model would have to sample from a uniform and independent probability distribution to guarantee that one of the guesses is correct. Thus, a perfect prediction would result in a per-word perplexity score of 1, and a perplexity score of 50 (for example) implies that the model thinks that about 50 different words are equally likely.

At the end of training, the mean per-word perplexity score of the 10 SRNs was 43.8 ± 0.05 (M ± SEM), and that of the 10 LSTMs was 42.6 ± 0.01 (M ± SEM). Compared to the same score before training (4102.2 ± 2.4 for SRN, 4095.9 ± 0.6 for LSTM), this is a significant reduction, and strong evidence that learning of word sequences has taken place. In other words, the average number of equally likely predictions across all words in the test data were reduced from approximately the total number of words in the vocabulary before training to only about 42–44 at the end of training for the SRN and LSTM. Because Skip-gram cannot predict word sequences there is no way to directly compare Skip-gram on this measure.

### Analyses of Semantic Structure

We performed several analyses to assess the semantic structure that the neural networks acquired. These can be divided into two categories reflecting the two theoretical questions being investigated. The first of these was the extent to which a model learned to represent semantic knowledge in terms of abstract semantic features, and the second was the extent to which semantic knowledge is hierarchically organized.

#### Encoding of Abstract Semantic Features

**FIGURE 2:**
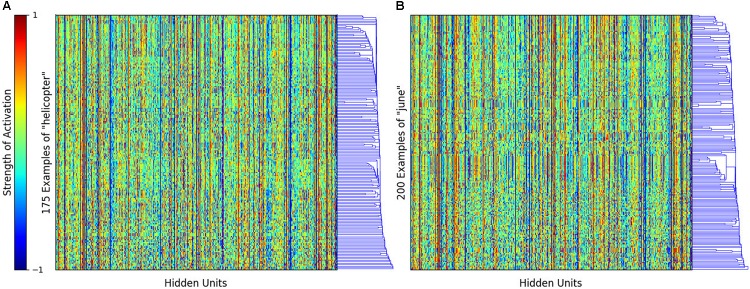
Heatmap showing the SRN hidden unit activations for the word **(A)** *helicopter* and **(B)** *june*. The rows represent the different occurrences of the word in the corpus, and the columns represent the 512 different hidden units. The rows are hierarchically clustered such that rows that are more similar are located together more closely.

To address the first question, we investigated the internal representations of the SRN. For example, **Figure 2** shows the hidden layer activations for the word *helicopter* and *june*. It is important to note that, for recurrent models like the SRN and LSTM, each occurrence of the word results in a different pattern of activations because of the influence of the prior context (i.e., the input coming into the hidden layer from its recurrent activation). Because there are 175 occurrences of helicopter in the training data, **Figure 2A** has 175 rows (one for each occurrence of the word) and 512 columns (one for each hidden unit in the SRN). The rows are hierarchically clustered such that rows that are more similar are located together more closely.

A number of interesting patterns emerge from this analysis. First, while each occurrence of *helicopter* generates a slightly different pattern because of the previous context, these patterns are overall quite similar, unsurprising because *helicopter* is a relatively unambiguous word in child-directed speech. This can be contrasted with the word *june*, where we can see clear polysemy (e.g., it is used as a name and to refer to a month) in the form of clusters of rows that are quite different from the rest. This Figure (and quantitative measures that can be derived from the data) nicely demonstrates that SRNs and LSTMs can accommodate and explain effects of polysemy and ambiguity.

**FIGURE 3:**
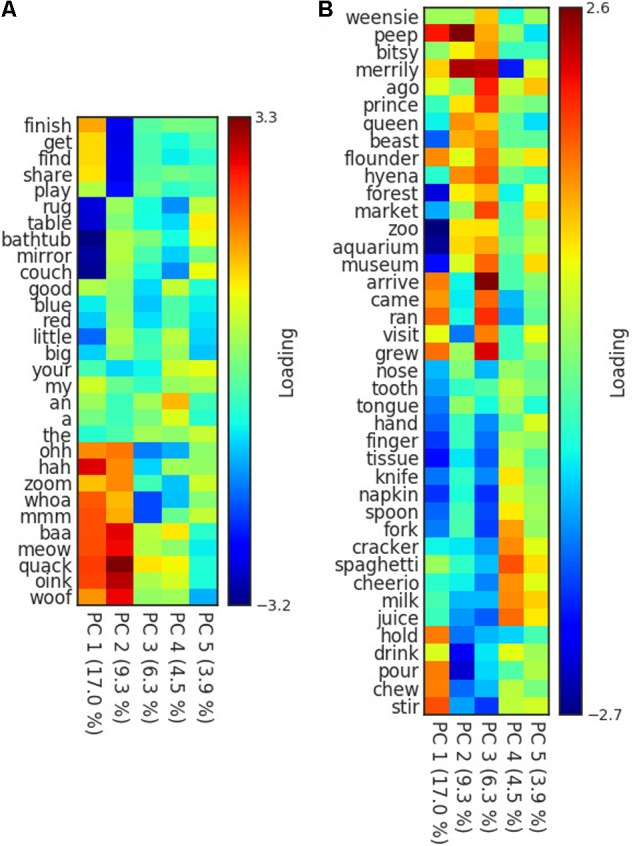
Heatmap of principal component loadings of words grouped by **(A)** grammatical category, and **(B)** activity context. Principal Components Analysis was computed using hidden layer activations learned by the SRN.

To address the question of whether these hidden units are coding for or representing abstract features or properties of the words, we performed a principal components analysis on the hidden units for all vocabulary words. We then assessed whether the principal components are meaningfully interpretable by tracking which words load heavily on a principal component. **Figure 3A** shows the extent to which selected words and categories load heavily on the first five principal components. In **Figure 3A**, we show five frequent words from a set of different grammatical categories (verbs, nouns, adjectives, function words, interjections, and onomatopoeia).

The initial two principal components are coding for high level, grammatical features that are understandably very important for predicting word order. The first principal component of the hidden layer activations (accounting for approximately 18% of the hidden layer’s variance), appears to be coding for something like whether a word is a noun (dark blue) vs. not a noun (dark red). But it is important to remember that what the neural networks are learning to do is predict word sequences, and this is best understood as the extent to which a word tends to follow words like ‘the’ and ‘my,’ or predict words like ‘is,’ ‘can,’ ‘was,’ and ‘have.’ The second principal component, accounting for an additional 13% of the variance, looks to be primarily distinguishing between verbs at one extreme (dark blue) and interjections and onomatopoeia at the other (dark red). Careful inspection of the loadings of all words in the in model suggests that this dimension is coding for whether a word tends to appear in isolation (onomatopoeia, interjections, and other similar words) or effectively cannot, as in the case of verbs that require nouns and other arguments.

After the first two principal components, the later components begin encoding semantic details. **Figure 3B** shows a different set of words that highlight that component three is effectively coding for the activity context, specifically whether the context is “eating,” compared to something more akin to “playing.” Nouns and verbs relating to playing, singing, reading, watching television, and the locations where those event occur, have highly positive activations, whereas nouns and verbs relating to eating have highly negative values on this component. This is not surprising as these are likely two of the most frequent and coherent events in young children’s lives, and are also orthogonal in the sense that they rarely occur together.

#### Structure and Organization of the Internal Representations

The second question was whether the model’s internal representations show signs of structure and organization. Given that we have just shown that the model is representing abstract semantic features of words, it is necessarily the case that some organization in terms of similarity structure must exist. But just how structured and organized is the information? As noted, the fact that the model represents each distinct occurrence of a word slightly differently demonstrates its ambiguity-representation capabilities. It does, however, raise the issue of the proper way to assess the similarity space across words, given the diversity of representations within words.

For the following analyses, we computed the pairwise similarity between all 720 probe-words and all 4096 words in the vocabulary in the following manner. First, we re-input the corpus into the model (after training and without updating the weights) and saved the hidden layer activations at the end of every sequence. To obtain a single word representation, we averaged all activations obtained for sequences in which the last word corresponded to the word of interest. We then obtained similarity scores by computing the correlations between these representations.

In the case of Skip-gram, the representation of a word was obtained simply from the weights connecting that word’s input unit to the hidden layer. For example, the input unit for *dog* has 512 weighted connections projecting to the 512 hidden units. Thus, to the extent to which two words make similar predictions about what their surrounding contexts are, they tend to have similar values for the weighted connections projecting to the hidden layer.

#### Overall Similarity between Models

Our next analysis addresses how similar the overall semantic spaces were in the 10 different randomly initialized runs of each model, and also how similar the overall semantic spaces were between models. In other words, if the first SRN thought *dog*’s similarity with *cat*, *shoe*, *cloud*, and *car* were 0.95, 0.76, 0.81, and 0.91, respectively, and the second SRN thought the scores were 0.94, 0.77, 0.80, and 0.90, this would reflect high agreement between the models. We computed this quantitatively as one would inter-rater reliability (Shoukri, 2010), by taking each model’s 720 by 4096 matrix of similarity scores and correlating them with one another, resulting in a *r*-value measuring the similarity of different models’ semantic spaces. These analyses showed that the different instances of the SRN were most similar to one another, (*r* = 0.967 ± 0.0002, mean ± standard error), that the LSTM instances were less similar (*r* = 0.959 ± 0.002), and that the different instances of Skip-gram were the least similar to one another (*r* = 0.889 ± 0.0005). Moreover, the semantic spaces of the SRNs and LSTMs were much more similar to each other (*r* = 0.92 ± 0.0004) than those of either the SRN and Skip-gram (*r* = 0.248 ± 0.0003) or the LSTM and Skip-gram (*r* = 0.284 ± 0.0005).

#### Nearest Neighbors

The first qualitative analysis we performed was to obtain a list of the most similar words (nearest neighbors in high dimensional semantic space) for each of the 720 probe-words. **Table 3** shows the five nearest neighbors for five probe-words for all three models. The words were chosen to illustrate typical semantic relations the model has acquired.

**Table 3:** Nearest semantic neighbors after training for 1 of the 10 models for selected words, in terms of the average hidden activation state of the network (for SRNs and LSTMs) and in terms of the weight matrix (for Skip-gram).

Dog	Bed	Shoe	Banana	Five
**SRN**				
Squirrel 0.95	Crib 0.93	Sock 0.97	Carrot 0.97	Six 0.95
Fox 0.95	Room 0.92	Sneaker 0.95	Pretzel 0.96	Four 0.95
Horse 0.95	Desk 0.92	Boot 0.95	Cracker 0.96	Three 0.94
Tiger 0.95	Pouch 0.92	Sandal 0.95	Cheerio 0.96	Ten 0.93
Wolf 0.95	House 0.92	Jacket 0.94	Lemon 0.96	Seven 0.93
**LSTM**				
Wolf 0.95	Desk 0.94	Sock 0.98	Cheerio 0.96	Four 0.97
Fox 0.95	Crib 094	Sneaker 0.96	Carrot 0.96	Six 0.96
Horse 0.95	Shade 0.93	Sandal 0.95	Pretzel 0.96	Eight 0.94
Mouse 0.95	Bedroom 0.93	Boot 0.94	Hamburger 0.96	Seven 0.94
Penguin 0.94	Room 0.93	Sweater 0.94	Peach 0.95	Three 0.94
**Skip-gram**				
Pup 0.76	Sleep 0.63	Sock 0.77	Pear 0.58	Six 0.88
Collie 0.62	Crib 0.59	Sneaker 0.77	Raisin 0.56	Four 0.83
Kitten 0.57	Blanket 0.54	Sandal 0.64	Frozen 0.55	Seven 0.77
Woggy 0.56	Bedroom 0.53	Pant 0.63	Cereal 0.55	Three 0.74
Bark 0.53	Nap 0.47	Shoelace 0.58	Oatmeal 0.54	Eight 0.69

A number of notable facts are worth pointing out about the nearest neighbors. First, the nearest neighbors of most probe-words share clear semantic relations, and this is evidence that these models have acquired general knowledge about semantic similarity, which strongly supports the distributional hypothesis. Second, this fact was true for all three models, with obvious qualitative differences in the relatedness or type of relatedness between a probe-word and its neighbors.

A third detail is that the there seems to be a general trend in the types of semantic relations the models thought were similar, as a function of the type of word. If the word is an example of a relatively well-defined or rule-based category, its neighbors tend to be members of the same category, even if such pairs do not co-occur in the corpus and thus are not likely to be thematic relations (such as *dog* and *tiger*). In contrast nearest neighbors for many human artifact categories (like ‘tools,’ ‘household rooms,’ and ‘furniture’), while still including mostly taxonomic relations, also include some thematically related neighbors. This pattern of data that shows an interesting resemblance to an observed bias by toddlers to use words to group taxonomically related things for well defined categories (Keil, 1992; Waxman and Markow, 1995). The model predicts this bias may be less strong for artifact categories than for natural kinds and rule-based categories like numbers, months, and days.

#### Dimensionality Reduction

**FIGURE 4:**
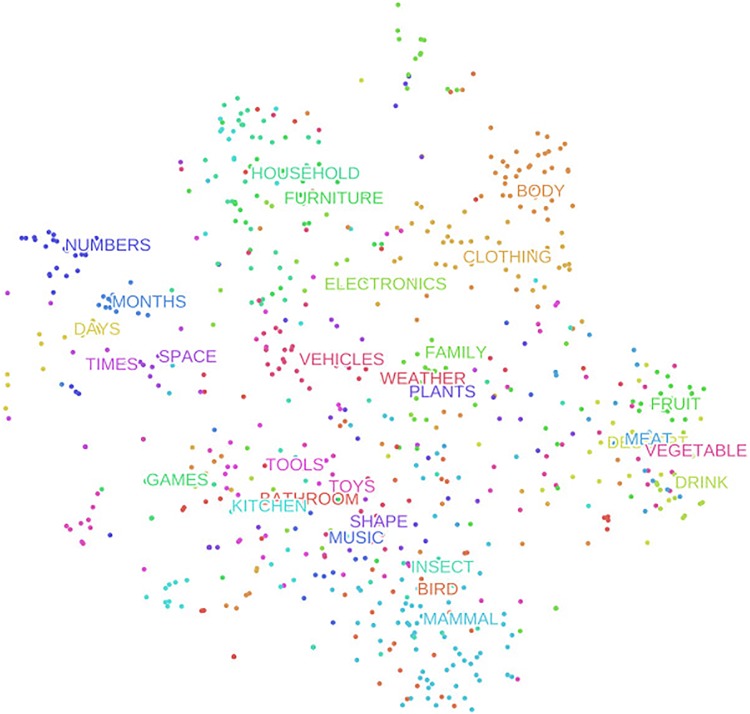
A t-SNE dimensionality reduction projection, showing a 2-D representation of the relative similarities of the 720 probe-words learned by the SRN.

In order to visualize distances between probe-word representations in the model’s 512-dimensional hidden activations space, we used a t-SNE dimensionality reduction algorithm (van der Maaten and Hinton, 2008), available via the Python package Scikit-Learn. We ran the algorithm using the average hidden activations for each probe-word as input (shown in **Figure 4**). Due to space constraints, we only show the t-SNE for a single SRN, because no significant qualitative differences existed between different instances of the model.

Several qualitative patterns emerged from this two-dimensional representation. When inspecting the category labels which are positioned at the average location across category members, an organizational scheme between categories becomes noticeable. First, words belonging to the categories ‘times,’ ‘months,’ and ‘days’ occupy a section of the similarity space that is distinct from all other probe-words. It makes sense that these are separate from items referring to concrete objects, and given that they all relate to timekeeping in some form, that they should be positioned closer to each other. Probe-words in the categories ‘bird,’ ‘insect,’ and ‘mammals’ also form a distinct super-category cluster in the bottom portion of the **Figure 4**. Categories containing non-living objects, and edible objects also occupy distinct portions of the similarity space.

Lastly, of note is the location of the probe-words belonging to the category ‘body’ in the top-right portion of **Figure 4**. It is obvious that body parts are not human-made objects like those referred to by words belonging to the categories ‘toys’ and ‘games,’ and that they aren’t living objects with agency either, so they are not close to ‘birds,’ ‘mammals,’ or even ‘family’ clusters. In fact, the ‘body’ category occupies its own distinct space along with ‘clothing,’ even though clothes are far from being the same kind of object, physically, as well as conceptually. However, when thinking about the relationship in terms of interactions taking place in the real world, their adjacency in **Figure 4** becomes understandable. The human body is dressed more often than anything else we might do to it. Notice also that probe-words belonging to the categories ‘kitchen’ and ‘bathroom’ are located separately from ‘household’ and ‘furniture.’ This spatial arrangement does not hold reliably when rerunning the t-SNE algorithm, which is not deterministic. While these two categories are often located more closely to ‘household’ and furniture,’ we show this arrangement because we think that the model is picking up on an important difference in the way objects in the kitchen and bathroom are used compared to those in other places of the household. We tend to interact more often with objects in the former two, either for cooking or tending to our hygiene. Objects in the ‘household’ and ‘furniture’ category, in contrast, are less frequently interacted with. The above two observations suggest that the model organizes objects not only taxonomically, but also pragmatically.

#### Similarities between Categories

**FIGURE 5:**
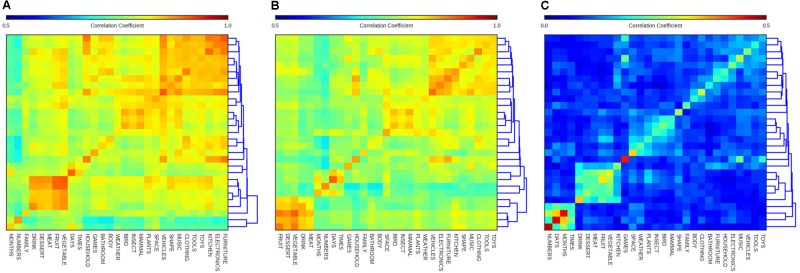
Dendrogram heatmap diagrams showing the average similarity of words (i.e., the Pearson correlations of the words’ hidden state activations) within and between categories for **(A)** SRN, **(B)** LSTM, and **(C)** Skip-gram. Row ordering mirrors that of columns. Note color scale is different for skip-gram.

To get a more complete understanding of the extent to which the model’s semantic structures are taxonomically-driven and hierarchically-structured, we constructed a dendrogram heatmap reflecting the similarities between probe-words within the same category and between different categories, shown in **Figure 5**. To do this, we started with a 720 by 720 matrix containing similarity scores for each pair of probe-words. Next, we removed the diagonal of this matrix (all ones, reflecting that each word was perfectly similar to itself), and then computed the average similarity of words within and between each of the 29 categories. This resulted in a 29 × 29 matrix of similarity scores. The rows and columns of this matrix were re-arranged by performing hierarchical clustering on the resulting 29 × 29 matrix. The resulting heatmap has higher values on the diagonal, indicating words in the same category have strongly correlated activation states. But in addition to this main effect of words being more similar, on average, to other words of the same category, there were also off-diagonal clusters which indicate cases where the model has learned a set of closely related categories. For example all of the categories containing ‘food’ probe-words are found in the lower left portion of **Figure 5**. A smaller cluster is obtained for categories containing living items, and another large grouping in the upper-most right portion of **Figure 5** includes categories containing non-living objects like ‘toys,’ ‘tools,’ and ‘vehicles’.

The dendrogram on the right side of each heatmap in **Figure 5** gives a sense of the hierarchical organization underlying the model’s similarity judgments. The categories ‘numbers,’ and ‘months’ occupy a distinct branch, indicating that these categories are used in a fairly distinct way (paralleling their distinct clusters in **Figure 4**). These results reflect an interesting property of the models. As shown in earlier work (Elman, 1990; Rogers et al., 2004), neural networks, while not explicitly encoding or representing hierarchical structure, nonetheless produce a set of activations whose similarity encodes hierarchical structure in a latent way. This fact remains true even when the input is noisy naturalistic data, where these patterns are not explicitly built into the model’s training data. The qualitative nature of these taxonomic and hierarchical structures presents an intriguing set of testable hypotheses, namely whether children acquire a semantic structure like that acquired by the model, and whether these structures are a quantitative and qualitative fit to behavioral data.

More clearly than in the neighborhood or t-SNE analyses, there do seem to be interesting differences between the three types of models, in terms of their between- and within-category relationships. Looking at the LSTM’s heatmap dendrogram in **Figure 5**, we noticed that the most separate cluster, determined by the dendrogram to the right, contains all the food categories, rather than time-related categories. Instead, the LSTM has a distinct fourth cluster for timekeeping categories, including ‘days’ and also ‘numbers,’ although the latter category can be more broadly used. **Figure 5** also shows the heatmap dendrogram for Skip-gram, which is different in many respects compared to the two recurrent neural networks. First, the similarities are globally lower, which is an artifact of the difference in the two training algorithms, rather than a difference in the global similarity structure. While the minimum and maximum similarities are shifted, we kept the size of the range the same across all diagrams to enable comparing relative similarities between categories across models. Another important difference is that the cluster of categories referring to human-made artifact categories is much less distinct. Indeed similarities within these categories are much higher relative to similarities across categories. One might conclude from this that Skip-gram has learned more about the differences in the probe-words referring to human-made objects than their similarities. Its four most prominent clusters include animal and food categories, as in the SRN, but also a time-keeping category cluster, as in the LSTM, and a unique cluster referring to objects or concepts typically found outside, including ‘space,’ ‘weather,’ and ‘plants.’ It is interesting that Skip-gram didn’t acquire a human-made categories cluster, but was able to cluster categories based on the concept of ‘outside.’ A final important difference to the recurrent neural networks is the grouping of ‘kitchen’ with the food categories instead of with its more taxonomically related categories ‘bathroom,’ ‘household,’ or ‘furniture.’ From this analysis, it has become clear that Skip-gram tends to group categories more thematically than the two recurrent neural networks.

”

#### Hierarchical Structure within Categories

**FIGURE 6:**
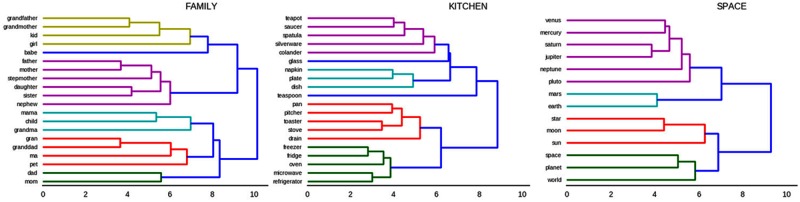
Hierarchical clustering dendrogram of words in the categories “family members,” “kitchen items,” and “space.

To demonstrate the extent to which the internal representations have latent hierarchical organization *within* each category, we used the same clustering algorithm as above, this time restricting the analysis to similarity scores from within one category. Because space is limited, **Figure 6** only shows examples of the categories ‘family,’ ‘kitchen,’ and ‘space from the SRN.

Many interesting details about the semantic structures learned by the models become apparent from these figures. Beginning with ‘family’ (**Figure 6**, left), the most closely related word pairs (i.e., pairs which share a branching point with the shortest distance from 0) are *grandfather* next to *grandmother*, and *father* nearest to *mother*. It is notable that synonym-like words for the same role do not tend to group together, instead grouping with their opposite-gender counterpart (*father* and *mother*, not *ma* and *mother*, are more closely related). This happens because contexts that follow the word *mother* tend to be very similar to those that follow the word *father*, resulting in the model learning that these two words are very similar or substitutable. Because the SRN is explicitly learning which words are substitutable with one another, it ends up with semantic organization that reflects contextual and pragmatic factors such as that *mother* and *ma* tend to be used in very different situations (predicted by formality), rather than a semantic organization that reflects dictionary definitions or feature-based synonyms. Again, this forms an intriguing testable hypothesis about the macro- and micro-organization of children’s semantic knowledge, namely that these pragmatic and contextual factors may play a larger role than has been supposed.

Similar insights are provided by the hierarchical clustering of words in ‘kitchen’ (**Figure 6**, middle). The largest two clusters seem to be separated according to objects used to prepare food (bottom cluster) and objects which are associated with the eating of food (top cluster). Microwaves and toasters, referred to by words in the bottom cluster, modify food by changing their temperature, whereas most words in the top cluster refer to objects that do not modify, but instead are present during consumption of food, such as teapots, silverware, napkins, etc.

The third clustering is illustrative because it shows that hypernyms such as ‘world,’ ‘planet,’ ‘star’ are distinctly separated from hyponyms, such as ‘venus,’ ‘mars’ (**Figure 6**, right). In other words, the hypernym-containing cluster on the bottom of the figure contains words that do not refer to any particular object in space, whereas those in the top cluster do. This provides evidence that the model can learn to separate between concrete objects and categories containing those objects.

#### Quantitative Analyses of Semantic Category Knowledge

Next we asked to what extent the internal representations can be used in a semantic classification task, in which two probe-words are judged to be in the same category. Judgments are based on a 720 by 720 matrix of the similarity of all probe words with one another. In this task, all word pairs’ similarity scores (S) were compared against a decision threshold and used to guess if the two words belonged to the same semantic category. We analyzed these results in a signal detection framework, computing hits, misses, correct rejections, and false alarms for each probe-word pair at multiple similarity thresholds (*r*, between 0.0 and 1.0 with step size 0.001). In other words, if two probe-words i and j belong to the same category, and *S*_i,j_ > *r*, a hit is recorded, whereas if *S*_i,j_ < *r*, a miss is recorded. On the other hand, if the two probe-words do not belong to the same category, either a correct rejection or false alarm is recorded, depending on whether *S*_i,j_ < *r* or *S*_i,j_ > *r*. For each probe-word, we calculated the sensitivity and specificity, and averaged the two to produce the balanced accuracy. This procedure eliminates bias resulting from the fact that a vast majority of word pairs do not belong to the same category. The measure of interest was the average of all the probe-words’ balanced accuracies at the similarity threshold which yielded the highest value.

We repeated this process for each of the 10 SRN, LSTM, and Skip-gram models to obtain an average balanced accuracy of 70.0% ± 0.05% (mean ± standard error) for the SRNs, 73.4% ± 0.05% for the LSTMs, and 73.7% ± 0.03% for Skip-grams. With such large differences between models and low variances within models, *t*-tests comparing differences between models result in very large differences: *t*(18) = 45.88, *p* < 0.0001, *r*^2^ = 0.992 for the difference between the SRN and LSTM; *t*(18) = 77.38, *p* < 0.0001, *r*^2^ = 0.997 for the difference between the SRN and Skip-gram; *t*(18) = 13.56, *p* < 0.0001, *r^2^* = 0.911 for the difference between the LSTM and Skip-gram.

In the case of the SRN, this means that on average a probe-word pair has a 70.0% chance of being correctly classified as belonging or not belonging to the same category. This is well above 50%, the score that an untrained model would be expected to receive. It is not surprising that both the LSTM and Skip-gram outperform the SRN on this task, given previous research demonstrating their improved performance on sequence learning and semantic tasks, respectively. It is somewhat surprising that Skip-gram achieved only a very small improvement compared to the LSTM, given that Skip-gram’s architecture is uniquely optimized to produce high quality word representations, whereas the LSTM’s objective is to learn sequential dependencies.

**FIGURE 7:**
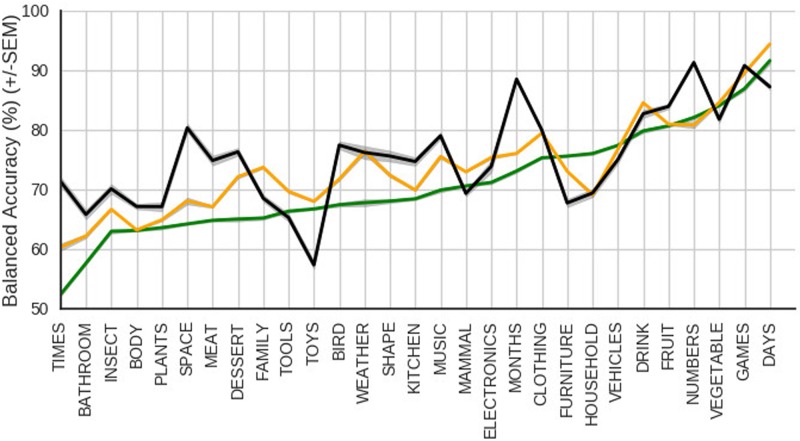
Balanced accuracy when using a model’s similarity scores to judge whether two words belong to the same or to different categories. The gray area represents the standard error of the mean. Colors: LSTM (orange), SRN (green), and skip-gram (black).

Inspecting the balanced accuracy for individual pairs sorted by category membership (shown in **Figure 7**), we found a large range across different categories, ranging from just above 50% (for words in the ‘times’ category, like *noon*, *minute*, *midnight*, *o’clock*), to just over 90% (for words in the ‘days’ category, like *monday*, *tuesday*, and *wednesday*). These differences are a quantitative assessment of which words’ internal representations form more cohesive categories, and which words the models would have difficulty determining belong to the same category. This is important because it allows us to make testable predictions about language development for future behavioral experiments. Comparing the balanced accuracy of the SRN to those of the other two models, reveals that the LSTM and Skip-gram achieve slightly better scores for almost all categories. While the performance profile by the LSTM follows closely that of the SRN, Skip-gram’s profile differs more dramatically (see spikes in **Figure 7** at categories ‘times,’ ‘space,’ ‘toys,’ ‘months,’ and ‘numbers’).

To better understand Skip-gram’s qualitative difference in performance, we compared nearest neighbors produced by all models for the categories ‘space’ and ‘months,’ two categories where Skip-gram clearly outperforms the recurrent models, and found distinct differences. The LSTM’s and SRN’s nearest neighbors to *earth* are generally locations, whereas Skip-gram’s neighbors feature planets more prominently, and few locations. A similar trend is noticeable for the word *planet*, for which Skip-gram’s nearest neighbors are exclusively other planets, and the LSTM’s nearest neighbors are words referring to things that can be found on a planet, such as a *country*, *sea*, *cloud*, etc. Furthermore, comparing nearest neighbors in the category ‘months,’ we found that the recurrent network models produced hardly any words referring to months for *may*, *march*, and *june*, whereas Skip-gram did. This is not surprising because *may* and *march* are frequent verbs, and *june* is a frequent name in the corpus. The recurrent neural networks seem to be less willing to group together words if the category is more abstract (in the case of ‘space’), or if its members have multiple meanings (‘months’). Learning outcomes, as with previous connectionist models, depend on how frequently and how consistently words are used (Seidenberg and McClelland, 1989). By performing worse on some items, the model is making specific predictions about how frequency and consistency may affect the categories and conceptual structure that children acquire, which can be tested in future research.

It is important to note that the categories were chosen by the authors and revised based on the explicit judgments of adult experimental participants. It could be the case that the categories with lower scores are less “real” in either a natural or psychological sense, and thus these lower scores reflect exactly how we would expect the model to perform. It could also be the case that these categories, while quite real to adults, are less important to children and thus not frequent or consistent in child-directed speech. Follow-up corpus analyses and behavioral experiments (with children and adults) can further investigate the natural or psychological reality of these categories, and assess the extent to which different models predictions about the cohesiveness of a category reflect the representations that children acquire.

#### Thematic and Taxonomic Bias

To investigate further the differences between the models, we generated nearest neighbors from three categories. Inspecting the neighbors that Skip-gram produced (**Table 4**, right column), we noticed a strong thematic bias. For example, Skip-gram’s nearest neighbors of ‘snow’ include ‘man,’ ‘white,’ and ‘melt’ which are not objects related to weather conditions, but instead are thematically associated with ‘snow.’ In contrast, neither of the recurrent neural networks (**Table 4**, left and middle columns) produced neighbors referring to the properties of snow such as its color, or its ability to melt. Nearest neighbors produced by the recurrent neural network instead appear to be constrained by being a noun; no other organization became apparent to us. More evidence for a thematic bias can be found by inspecting Skip-gram’s neighbors for the word ‘fish,’ which include ‘swim,’ which describes a property of fish, and ‘glub,’ a sound produced by fish (or at least, the way that such sounds are described in interactions with children). In contrast, the LSTM and SRN produced nearest neighbors that refer to fish-like objects while none refer to properties of fish. These differences is consistent with previous research, which has shown that models strictly tracking co-occurrences between words within a window tend to define words’ similarities in terms of their substitutability, whereas models tracking co-occurrences between words in a document (and thereby disregarding word order information) tend to define word similarities in terms of more thematic-like relationships (Rubin et al., 2014). Because the number of times Skip-gram is trained to associate a word with another word in its context is proportional to the distance between the two words, Skip-gram does have access to word order information; however, access to this information is limited compared to models that are trained to learn word order explicitly (SRN and LSTM). This partial loss of word order information might account for Skip-gram’s greater propensity for generating thematically related neighbors.

**Table 4:** Nearest semantic neighbors from SRN, LSTM, and Skip-gram for two words in the categories ‘weather,’ ‘meat,’ and ‘months.’

SRN	LSTM	Skip-gram
**Snow**	**Snow**	**Snow**

Treasure 0.87	Rocket 0.90	Man 0.57
Log 0.86	Fish 0.88	Flake 0.55
Motorcycle 0.86	Snail 0.88	White 0.52
Taxi 0.86	Cloud 0.88	Baum 0.50
Mail 0.86	Mole 0.88	Melt 0.46

**Rain**	**Rain**	**Rain**

Flash 0.92	Dark 0.91	Spout 0.49
Dust 0.91	Daytime 0.90	Outside 0.46
Land 0.90	Dust 0.89	Bitsy 0.45
Steam 0.90	Steam 0.88	Itsy 0.45
Crowd 0.90	Colder 0.88	Spider 0.44

**Meat**	**Meat**	**Meat**

Salad 0.97	Broccoli 0.96	Soup 0.57
Bread 0.97	Oatmeal 0.95	Carrot 0.56
Pizza 0.96	Salad 0.95	Broccoli 0.55
Oatmeal 0.96	Bread 0.95	Cheese 0.53
Cereal 0.96	Macaroni 0.95	Vegetable 0.53

**Fish**	**Fish**	**Fish**

Whale 0.91	Penguin 0.92	Angler 0.53
Hay 0.91	Snail 0.91	Turtle 0.49
Goldfish 0.91	Goldfish 0.91	Glub 0.48
Goose 0.91	Whale 0.91	Swim 0.46
Turkey 0.91	Bug 0.91	Fins 0.46

**April**	**April**	**April**

Buster 0.93	Harvey 0.93	Fifth 0.59
Harvey 0.93	Darling 0.93	February 0.56
Hank 0.93	Abba 0.93	Twenty 0.53
September 0.93	Correct 0.92	Saturday 052
January 0.93	America 0.92	October 0.51

**Month**	**Month**	**Month**

Year 0.97	Year 0.97	Year 0.80
Degree 0.93	Thousand 0.92	Week 0.68
Ounce 0.93	Hour 0.92	Twenty 0.64
Dollar 0.93	Week 0.92	Ounce 0.58
Thousand 0.92	Hundred 0.92	Thirty 0.56

## Discussion

We tested the hypothesis that a distributional learning mechanism might account for the acquisition of useful semantic structure from noisy naturalistic language input. This proposal has a long history, but until recently it has been difficult to evaluate how effective such a process might be in the development of the human semantic system, and how much knowledge and the extent of the knowledge and structure for which it could account. However, due to increasingly more powerful and sophisticated computational models, large naturalistic datasets, and computer power, this hypothesis can now be tested on a large scale. To this end, we trained three different neural networks (SRNs, LSTMs, and Word2Vec’s Skip-gram) on over 5-million words of child-directed speech and examined both qualitatively and quantitatively the semantic structure underlying the representations that emerged for 720 probe-words. We compared the results to better understand the advantages and limitations of each model.

We found that all three neural network models learned complex semantic relationships, demonstrating learning of complex internal representations such as those found in the models of Elman (1990) and Rogers et al. (2004). This research shows that the principles demonstrated by those models do not depend on the cleanliness of their artificial datasets. To the contrary, this work shows that the structure of the input that children receive is in fact highly organized and capable of supporting learning in neural or cognitive system without strong priors about the organizational structure that might be learned.

Our second purpose was to address whether specific instantiations of these neural network models can be better characterized as fitting the “child as data analyst” metaphor, or the “child as theorist” metaphor, as outlined by Waxman and Gelman (2009). Across a range of analyses, we showed that these neural network models are going far beyond representing simple statistical associations between words, and are instead representing word meanings in terms abstract features that group words into taxonomic and hierarchically-organized structures. Hidden units in neural networks, when there are fewer hidden units than input or output units, can be thought of as a procedure for discovering abstract features that allow for efficient and organized representation of information.

We investigated whether the semantic structures the models acquired reflect the semantic structures that children acquire. The words that the recurrent neural network models considered to be the most similar tended to be *taxonomically* related (i.e., from the same category) rather than *thematically* related (i.e., co-occurring within the same situation or event). For example, it was very rare for those models to group semantically related words that were different parts of speech (*bounce* and *ball*, for example). This was true for the two recurrent models (the SRN and LSTM), because the stronger grammatical constraints (predicting word order) on these models led to more grammatically constrained semantic neighborhoods. Skip-gram, on the other hand, is less constrained by the grammar inherent in the input, and therefore produces less taxonomically constrained neighbors. The bias in the recurrent neural networks toward taxonomic relations is notable, given that children have been shown to assume that noun labels refer to groups of kinds of things, rather than groups of words that co-occur within a situation or event (Waxman and Markow, 1995). All three models also appeared to learn a latent hierarchical structure, capable of producing the kinds of behavior observed in children (Keil, 1992). Words tended to be most similar to other words of the same category, and then most similar to words belonging to a superordinate category.

We also investigated whether different neural network architectures (and the different theories of learning and memory that they represent) perform qualitatively or quantitatively differently with regard to their ability to learn semantic information. We found that, Skip-gram’s representations differed from those learned by the recurrent models by displaying a greater bias for learning thematic relationships. Furthermore, aligning with previous research, we found that Skip-gram and LSTM models tended to be slightly better in quantitatively evaluated situations (such as predicting whether two words belong to the same category) than the SRN. However, there is a very important caveat to mention here. This task is arguably artificial, and even if real, we do not have data on how children or adults would perform on a similar task. The higher performance reached by LSTM and Skip-gram on this task is evidence these are better machine learning models, but not that they are better models of human cognition. Furthermore, it is not clear whether the slight performance increase is important, given that we do not know whether the magnitude of this difference is in the range of variability between human subjects. Considering the relative architectural simplicity of the SRN, and absence of any human data, the SRN should by no means fall out of favor for cognitive scientists studying semantic memory. In the contrary, we think that its achievements make it all the more promising as a model of semantic development. Ongoing work is investigating how children perform on a number of semantic tasks, and these models will serve as sources of quantitative and qualitative predictions of children’s performance in those tasks.

One particularly important distinction in our work is the distinction between sequence prediction and semantic classification. Our primary interest in this paper was in modeling semantic development, and showing how semantic classification can emerge from sequence prediction. A model that learns to predict sequences must, by its very nature, learn which items tend to be substitutable, which is arguably what one needs to do in order to perform categorization behaviors. However, these two tasks, while mutually supporting one another to an extent, may compete with one another in the limit. Perfect (or as perfect as possible) sequence prediction on new input involves needing to learn abstract generalizations in the model’s hidden layer that can predict new situations. The abstractions necessary for doing this in language are very likely not identical to the abstractions necessary for interacting with world knowledge (Willits et al., 2015). As such, one could view getting very good at predicting language sequences as a case of overfitting to the specifics of that task or goal, resulting in a slight decrease in performance when classifying real world objects and events. As a consequence, models like Skip-gram which do not perfectly capture word order, may be providing a slight protection against such overfitting, resulting in slightly better semantic performance.

Many questions, limitations, and future research directions remain. The most obvious is the extent to which these models correctly predict behaviors about semantic development, beyond those qualitative matches we have shown here. In addition to further experimental validation, there remain follow-up analyses about the specific semantic structures of the models. Can the precise extent to which models develop taxonomic or hierarchical representations be quantified, and used to adjudicate between models? How do the models respond to perturbations in the input, and how do differences in quantity and quality of input, or learning more than one language at once affect acquisition of semantic knowledge?

Other questions involve the cognitive and neurobiological plausibility of the proposed learning and representational mechanisms. While models that are capable of capturing a greater number of the statistical properties of language exist (LSTM), we must keep in mind that language comprehension is a demanding process, and requires decoding of highly structured input within very short time (Friederici, 2002; Pylkkänen and Marantz, 2003). It is unlikely that humans use all the statistical information available in the input, as such a learning mechanism would be costly in terms of the expenditure of neural resources. Many language statistics contain overlapping information, and many may not be relevant to language comprehension at all. Moreover, language learning typically occurs well before the brain has achieved maturity, so developmental constraints further limit the learning mechanism. While it is tempting to speculate which of the three architecture most closely aligns with neurocognitive principles, there is no evidence to date to guide such speculations. Neither can we claim to have singled out a specific learning mechanism operating in children; rather our intention is to demonstrate the different kind of learning results taking place in different models.

A final limitation of note, but a very important one, is the fact that these models of semantics contain only linguistic input, and have no access to or knowledge of grounded, embodied, world knowledge that most (but not all) children receive from vision, hearing, touch, taste, and smell. Much ink has been spilled on the issue of what precisely distributional models are missing by not including this information (de Vega et al., 2008; Johns and Jones, 2012; Jones et al., 2015; Willits et al., 2015). While many researchers treat distributional models and grounded, embodied models as necessarily opposed, this need not be the case. There is no reason that a distributional model cannot operate over extra-linguistic, grounded world knowledge. In fact, Sadeghi et al. (2015), in a paper titled “You shall know an object by the company it keeps” used a distributional model operating over pictures (rather than words), and showed that such a model can learn the similarity structure of objects. Like with words, this makes sense, as the kinds of visual contexts that cats and dogs tend to occur in are similar to each other, and different from the kinds of contexts that forks and spoons occur in, or that shoes and socks occur in. Distributional similarity is an algorithm that can operate on any modality. Given the lack of non-linguistic data, it is actually quite impressive how well the recurrent neural networks learn to represent semantic content. But of course a more complete model of semantics employing the distributional hypothesis would include both linguistic and extralinguistic information, and the interaction between the two (Willits et al., 2015). In future work, we would like to extend the recurrent neural networks to include distinct auditory and visual layers that take audio and video input and learn to combine them into a composite representation. In the meantime, it may not be appropriate to call the type of learning taking place in models without extra-linguistic input as ‘semantic.’ We are optimistic that this issue is one of terminology, and does not relate to fundamental restrictions inherent in the learning algorithms employed in this study.

Our modeling results show that complex and highly organized semantic structure emerges automatically from learning the statistical regularities of child directed speech, supporting the idea that a neural network-like model of statistical learning might explain aspects of semantic development. We hope that further computational modeling efforts will continue to combine realistic models of children’s input with cognitively realistic models to contribute to our understanding of a wide range of phenomena in semantic memory and its development.

## Author Contributions

PH and JW worked jointly on the theory, research questions, and writing of the paper. PH performed the modeling simulations described in the paper.

## Conflict of Interest Statement

The authors declare that the research was conducted in the absence of any commercial or financial relationships that could be construed as a potential conflict of interest.
